# Plasmid-Coded Linezolid Resistance in Methicillin-Resistant *Staphylococcus aureus* from Food and Livestock in Germany

**DOI:** 10.3390/antibiotics11121802

**Published:** 2022-12-12

**Authors:** Tobias Lienen, Mirjam Grobbel, Bernd-Alois Tenhagen, Sven Maurischat

**Affiliations:** German Federal Institute for Risk Assessment (BfR), Department of Biological Safety, 10589 Berlin, Germany

**Keywords:** MRSA, linezolid resistance, food, livestock

## Abstract

Resistance of methicillin-resistant *Staphylococcus aureus* (MRSA) from food and livestock to last resort antibiotics such as linezolid is highly concerning, since treatment options for infections in humans might be diminished. Known mechanisms of linezolid resistance include point mutations in the 23S rRNA gene and in the ribosomal proteins L3, L4 and L22 as well as an acquisition of the *cfr*, *optrA* or *poxtA* gene. The objective of our study was to characterize antimicrobial resistance (AMR) determinants and phylogenetic relationships among linezolid-resistant (LR-) MRSA from food and livestock. In total, from more than 4000 incoming isolates in the years 2012 to 2021, only two strains from 2015 originating from pig samples exhibited linezolid resistance in the antimicrobial susceptibility testing with MICs of ≥8 mg/L. These LR-MRSA were characterized in detail by whole-genome sequencing and phylogenetic analyses using cgMLST. The LR-MRSA strains showed resistances to ten and eight different antibiotics, respectively. Both strains harbored plasmid-coded *cfr* genes mediating the linezolid resistance. The *cfr* genes showed identical sequences in both strains. In addition to the *cfr* gene, genes for phenicol and clindamycin resistance were detected on the respective plasmids, opening the possibility for a co-selection. The LR-MRSA differed distantly in the phylogenetic analyses and also to other MRSA from pig samples in the year 2015. In conclusion, the occurrence of LR-MRSA in food and livestock seems to be very rare in Germany. However, carriage of plasmids with linezolid resistance determinants could lead to further linezolid-resistant strains by horizontal gene transfer.

## 1. Introduction

Methicillin-resistant *Staphylococcus aureus* (MRSA) are frequently detected in livestock and food [1,2]. In addition to their resistance to virtually all beta-lactam antibiotics, resistance to critically important or last resort antibiotics such as linezolid is highly concerning for animal and human health due to the further reduction of treatment options for severe MRSA infections. Linezolid belongs to the oxazolidinone antibiotic group. Its mode of action is the inhibition of protein synthesis by binding to the ribosomal RNA [3]. Linezolid resistance may be mediated by several genes (*cfr*, *optrA* and *poxtA*) as well as by point mutations in the genes for the 23S rRNA and ribosomal proteins L3, L4 and L22 [4,5,6,7,8]. The *cfr*, *optrA* and *poxtA* genes are often located on plasmids and may be transmitted between staphylococcal species or even between different genera [9]. As well as mediating linezolid resistance, the *cfr* gene, for which the variants *cfr*, *cfr*(B), *cfr*(C), *cfr*(D) and *cfr*(E) were detected [9], may also cause resistance to phenicols, lincosamides, pleuromutilins, and streptogramin A [5]. *poxtA* is a phenicol–oxazolidinone–tetracycline resistance gene [7], whereas the *optrA* gene confers resistance to oxazolidinone and tetracycline antibiotics [6]. In addition, for the *optrA* gene, different variants have been discovered in enterococci [10]. Several studies have reported linezolid-resistant (LR-) MRSA strains in humans, livestock and food; however, the prevalence was rather low compared to resistance to other antibiotics [11,12,13,14]. In the One Health context, handling livestock or food has been shown to be a risk factor for the acquisition of livestock-associated MRSA from animals [15,16,17,18]. Therefore, it has to be considered that acquisition of LR-MRSA from animals might lead to difficult-to-treat infections in people working in the livestock or food sector. As a consequence, monitoring of antimicrobial resistance in MRSA from food and livestock is of high importance.

Knowledge about AMR genes and their detection and characterization by whole-genome sequencing (WGS) is of high value for medical treatment of MRSA infections. The prediction of AMR by sequencing may support faster treatment decisions for MRSA infections in comparison to cultural approaches. Moreover, phylogenetic relationships and transmission of resistance genes may be unraveled by analyzing and comparing whole genome sequences of MRSA. For instance, MRSA spread in livestock farms may be tracked genomically by the application of WGS and subsequent sequence analyses [19,20,21].

The aim of our study was to characterize LR-MRSA from food and livestock in Germany by WGS and phenotypic antimicrobial susceptibility testing. The genomic causes for linezolid resistance were discovered. Moreover, phylogenetic relationships among the strains were analyzed to evaluate the relationships of LR-MRSA from livestock and food.

## 2. Results

### 2.1. Genotypic Analyses and AMR

The strain collection of the National Reference Laboratory for coagulase-positive Staphylococci, including *Staphylococcus aureus*, at the German Federal Institute for Risk Assessment was screened for MRSA strains that were phenotypically linezolid-resistant. From more than 4000 incoming MRSA strains in the years 2012 to 2021, only two MRSA strains showed phenotypic resistance to linezolid, exhibiting a minimum inhibitory concentration of ≥8 mg/L. Both LR-MRSA strains harbored a SCC*mec* type V element, but carried different *spa* types t011 and t034. Accordingly, the strains were associated to the sequence type (ST) 398 that is highly prevalent in livestock in Germany. The LR-MRSA strains showed a multidrug-resistant phenotype with resistance to ten or eight of the included antimicrobials, respectively. Both LR-MRSA strains exhibited resistance to chloramphenicol, clindamycin, cefoxitin, linezolid, penicillin, quinupristin-dalfopristin, tetracycline and tiamulin. One of the two strains (LR-MRSA-1) was additionally resistant to streptomycin and trimethoprim.

**Figure 1:**
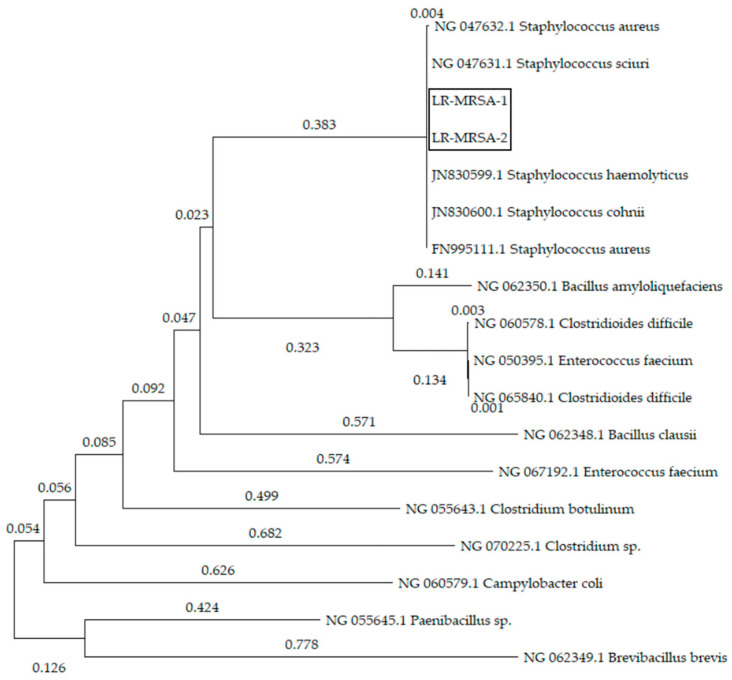
Neighbour-joining tree including evolutionary distance based on *cfr* gene sequences from LR-MRSA strains (marked in box) and various other bacterial species.

Point mutations in the 23S rRNA gene as well as in the ribosomal proteins L3, L4 and/or L22 genes were not discovered in the LR-MRSA strains. In both strains, a *cfr* gene was detected, whereas *optrA* or *poxtA* genes were not found. Gene sequences of the *cfr* genes were identical and clustered closely together with *cfr* gene sequences from other staphylococcal species in the NCBI database (Figure 1). In addition to the *cfr* gene, the LR-MRSA-1 strain harbored the AMR genes *ant(9)-Ia*, *dfrG*, *fexA*, *lnu*(B), *lsa*(B), *lsa*(E), *mecA*, *str*, *tet*(38), *tet*(K) and *tet*(M) whereas the LR-MRSA-2 strain additionally harbored the AMR gene *blaZ* but lacked *lsa*(B) and *str*.

**Figure 2:**
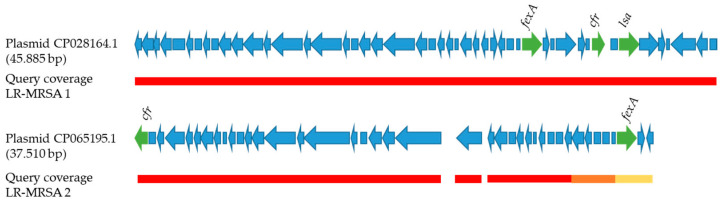
Illustration of gene arrangements on reference plasmids CP028164.1 and CP065195.1. AMR genes *cfr*, *fexA* and *lsa* are marked in green. Sequences of LR-MRSA-1 and LR-MRSA-2 showed high identities and coverages with regard to known plasmids. Color of bars represents sequence identity (red, ≥99%; orange, ≥97%; yellow, ≥96%).

Analyses of the flanking regions of the *cfr* genes revealed that the *cfr* genes were located on plasmids (Figure 2). The LR-MRSA-1 strain harbored sequences that were highly similar to the plasmid CP028164.1 (sequence identity 99.99%, query coverage 100%), whereas the LR-MRSA-2 strain showed sequences with a high identity to the plasmid CP065195.1 (sequence identity 98.73%, query coverage 95%). Both plasmids additionally harbored a *fexA* gene. Moreover, plasmid CP028164.1 carried an *lsa* gene.

### 2.2. Phylogenetic Analysis

**Figure 3:**
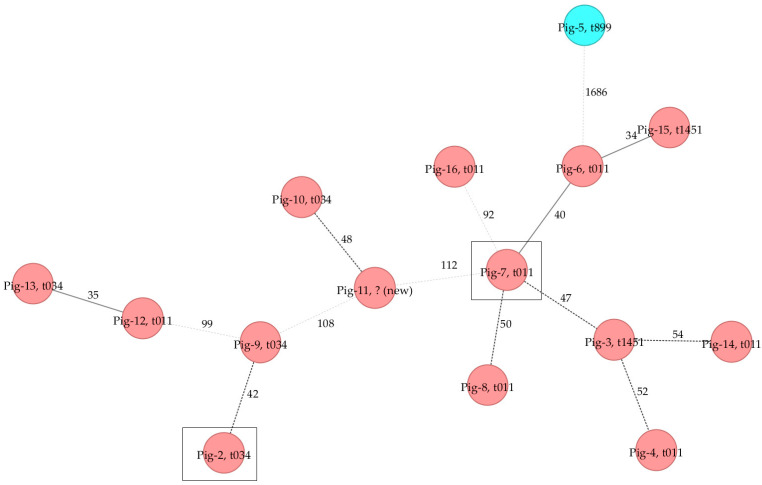
Minimum spanning tree including *spa* types based on cgMLST analyses of MRSA strains from pig origin from the year 2015. MRSA belonging to ST9 is illustrated in blue circle and MRSA ST398 in red circles. LR-MRSA strains are represented by Pig-2 and Pig-7 (marked by black boxes).

Core genome multilocus sequence typing (cgMLST) with concurrent minimum spanning tree (MST) analysis was carried out with the LR-MRSA strains in comparison to 13 other ST9- and ST398-MRSA strains with a pig origin and from the year 2015 to evaluate possible phylogenetic relationships and transmission events between strains from the same year and origin. The MST illustrates the high diversity of the MRSA strains in the year 2015 with distantly related strains (Figure 3). The two detected LR-MRSA strains were also distantly related.

## 3. Discussion

The risk of a lack in treatment options for infections caused by MRSA is ever-present, since resistance to a high number of antimicrobials, including critically important antibiotics, is occasionally detected in MRSA strains. Linezolid is one of the last resort antibiotics. Although it is not licensed for use in veterinary medicine, LR-MRSA have been found in livestock and food samples. As a consequence, transmission of these strains to humans via animal contact or food may occur and subsequent infections caused by these strains might be difficult-to-treat.

In our study, LR-MRSA obtained from food and livestock samples in Germany collected between 2012 and 2021, were analyzed in detail by WGS and phenotypic antimicrobial susceptibility testing. Only two LR-MRSA isolates were recovered from the strain collection. Both strains originated from pigs sampled in 2015. The two strains belonged to the livestock-associated ST398. In relation to the total number of MRSA, which were analyzed by our laboratory in this time period (>4000), the LR-MRSA only comprise a very minor fraction of MRSA. This is in line with recent data about a low proportion of LR-MRSA-caused human bloodstream infections in Europe [22] and the low global prevalence of LR-MRSA in clinical samples [11].

Linezolid resistance may be associated with point mutations in the 23S rRNA gene, mutations in the genes of the ribosomal proteins L3, L4 and L22 and/or with an acquisition of the *cfr*, *optrA* or *poxtA* gene, which are often harbored on mobile genetic elements [8]. In our study, point mutations in the 23S rRNA gene or in the proteins L3, L4 or L22 genes were not found in the detected LR-MRSA strains. Accordingly, a recent study from Belgium reported, in particular, the presence of *cfr*, *optrA* and *poxtA* genes in linezolid-resistant staphylococcal and enterococcal strains from food-producing animals [14]. Moreover, LR-MRSA harboring a *cfr* gene were recently detected in pigs in Spain [13] and in retail food in China [23]. In addition, a *cfr* gene was found in staphylococci from veal calves and pigs from farms in Germany and Luxembourg [24]. In our study, both LR-MRSA strains harbored a *cfr* gene. The *cfr* gene may be located on plasmids and thus be transmittable between staphylococci [9]. Plasmid-coded *cfr* genes were also found in the two strains of our study. In contrast, the genes *optrA* and *poxtA*, which may also mediate linezolid resistance and are often found on mobile genetic elements, were not detected in the strains of our study. Accordingly, in other studies, linezolid resistance in staphylococci from several food-producing animals or pigs was indeed mediated by a *cfr* gene, whereas *optrA* and *poxtA* genes were not detected [14,25]. Both *cfr* genes carrying LR-MRSA strains in our study exhibited a rather high MIC for chloramphenicol (>64 mg/L). This was most probably caused by two different resistance mechanisms that are transmitted by the *cfr* and *fexA* genes present in both isolates. As well as linezolid resistance, the *cfr* gene was described to confer resistance to phenicols, lincosamides, pleuromutilins, and streptogramin A antibiotics [5]. Moreover, the *fexA* gene mediates phenicol resistance as well. Both genes, *cfr* and *fexA*, were co-located in close proximity on a plasmid. In addition, the strains exhibited resistance to clindamycin, which belongs to the lincosamides, and tiamulin, a pleuromutilin. Both resistances may also be conferred by the *cfr* gene.

Linezolid is not licensed for use in livestock. Therefore, the question about the selective pressure for the acquisition and persistence of linezolid resistance determinants in livestock arises. Likely, LR-MRSA harboring resistance to other antibiotics that are used in livestock farming are selected by antibiotic use on livestock farms. Florfenicol may be used in pig farming for acute respiratory illnesses and this antibiotic was also used on German pig farms in 2015 [26]. Since the *cfr* and *fexA* gene confer a rather high MIC for florfenicol, use of florfenicol might have co-selected for linezolid resistance. Moreover, tiamulin may be used in pig farming in the treatment of dysentery. Tiamulin resistance is also transmitted by the *cfr* gene, so that a possible co-selection also might have appeared due to the use of tiamulin. In addition, the lincosamide lincomycin was used in pig farming in Germany in 2015 [26]. Both LR-MRSA strains harbored *lsa* and *lnu* genes, which may confer resistance to lincosamides. At least in the case of one LR-MRSA strain, the *lsa* gene was located on the same plasmid as the *cfr* gene. Therefore, use of lincomycin might have been another possibility for co-selection of the respective LR-MRSA strain. However, since resistance to florfenicol is coded on the same plasmid as resistance to linezolid, it is most reasonable to assume that florfenicol use was the main driver of co-selection after acquisition of the plasmids by the two LR-MRSA strains in our study. Moreover, it has to be considered that the detected LR-MRSA strains originated from retail and slaughterhouses. Therefore, a transmission of the strains by humans during animal and meat processing cannot be completely excluded. Hypothetically, workers carrying a linezolid-resistant strain in their noses or on their skin might have passed the strain to the meat.

In particular, the multidrug resistance phenotype of the two LR-MRSA strains, exhibiting resistance to ≥8 antimicrobial substances, is concerning with regard to treatment options for severe infections in animals and humans. This underlines the need to monitor AMR in MRSA from livestock and food. The introduction of such strains into hospitals would further reduce treatment options for severe infections in humans. Moreover, MRSA infections in animals, such as mastitis in cattle, might become difficult to treat, causing high costs for farm operators.

The application of WGS also allows for analyses of phylogenetic relationships among bacteria and typing methods based on WGS were shown to be suitable for tracking foodborne outbreaks [27]. The diversity of MRSA with a pig origin in our study was high and both LR-MRSA strains were only distantly related. MRSA belonging to ST398 or ST9 were clearly separated by cgMLST analyses. Interestingly, large allelic differences between the analyzed MRSA ST398 strains were observed in the MST although some strains harbored the same *spa* type, such as t011 or t034. Therefore, when investigating transmission of multidrug-resistant MRSA, *spa* typing alone might be misleading due to an insufficient discriminatory power. The improved discriminatory power of cgMLST analyses improves the traceability. The suitability of core genome analyses was also shown in other studies such as those on transmission of MRSA between pigs and humans in Australia [19], phylogenetic tracking of MRSA at German dairy farms [21] and the prevalence and diversity of *S*. *aureus* in the dairy value chain in Zambia [28].

To date, monitoring of MRSA in food and livestock is voluntary in Europe. Therefore, only a few European countries report on the prevalence of MRSA and their resistance phenotypes in livestock and food [29]. Our study illustrates that surveillance of AMR in MRSA from food and livestock in particular with regard to antimicrobials of great importance to the medical sector is an important contribution to the one health assessment of the resistance situation in *S. aureus* and for an early detection of MRSA with a high public health importance. Molecular surveillance based on sequencing may identify possible transmission events and the distribution of specific clones across matrices including animals, food and humans, such as farm staff.

## 4. Materials and Methods

### 4.1. Sampling and MRSA Strain Selection

From 2012 to 2021, the National Reference Laboratory for coagulase-positive Staphylococci, including *Staphylococcus aureus*, at the German Federal Institute for Risk Assessment obtained more than 4000 MRSA isolates. Two LR-MRSA strains (MIC ≥ 8 mg/L) were detected in the strain collection. Both LR-MRSA strains were collected in the year 2015 and originated from pigs (pig meat at retail or carcass from a slaughterhouse).

### 4.2. WGS and Bioinformatics Analyses

MRSA strains were inoculated in 5 mL brain–heart-infusion broth and incubated at 37 °C for 24 h. DNA of 1 mL culture was extracted using the Qiagen DNeasy Blood and Tissue Kit (Qiagen, Hilden, Germany) according to the manufacturer’s protocol modified by adding 10 µL lysostaphin (Sigma Aldrich, St. Louis, MO, USA) to the lysis buffer. The DNA library was prepared using an Illumina Nextera DNA Prep kit (Illumina Inc., San Diego, CA, USA) and the 150 bp paired-end sequencing run was performed on an Illumina NextSeq 500 instrument. Raw Illumina reads were trimmed and de novo assembled with the in-house developed AQUAMIS pipeline [30]. Bacterial characterization was carried out with the in-house developed Bakcharak pipeline (https://gitlab.com/bfr_bioinformatics/bakcharak, accessed on 1 October 2022) using the NCBI AMRfinder database [31] for screening of AMR genes. Moreover, software tools from the Center for Genomic Epidemiology (https://cge.food.dtu.dk/services/, accessed on 1 October 2022) were applied. ResFinder 4.1 [32,33,34] was used for point mutation investigation, SCCmecFinder 1.2 [34,35,36] was used for analyses of SCC*mec* types and spaTyper 1.0 [37] was used for determination of *spa* types. Analyses by cgMLST as well as MLST determination were conducted using Ridom SeqSphere+ version 7.0.4 according to *S*. *aureus* cgMLST scheme comparing 1861 alleles. Determination of point mutations in the ribosomal protein L3, L4 and L22 genes was conducted by alignment to reference sequences from the strains JP080 (AP017922.1) and M12/0145 (KU510541.1) using blastn suite (NCBI BLAST). Comparison and alignment of *cfr* gene sequences was conducted in MEGA X version 10.1.7. The *cfr* gene sequences of different bacterial species were retrieved in NCBI Genbank. Identification of plasmids was carried out in NCBI BLAST by using contigs with *cfr* gene carriage and sequence analyses of flanking regions.

### 4.3. Antimicrobial Susceptibility Testing

Antimicrobial susceptibility testing was performed by broth microdilution according to international guidelines (ISO 20776-1:2006 [38]/CLSI M07 Ed 11 [39]). It was carried out using a commercial standardized antibiotic panel (EUST scheme from Thermo Fisher Scientific, Waltham, MA, USA) that is recommended by the European Food Safety Authority (EFSA) for resistance monitoring in MRSA from livestock and food [40]. For interpretation of the minimum inhibitory concentration (MIC) of the individual strains, the EUCAST ECOFFs for *S*. *aureus* fixed in this EFSA technical specification [40] were used: penicillin > 0.125 mg/L; cefoxitin > 4 mg/L; chloramphenicol > 16 mg/L; ciprofloxacin > 1 mg/L; clindamycin > 0.25 mg/L; erythromycin > 1 mg/L; fusidic acid > 0.5 mg/L; gentamicin > 2 mg/L; kanamycin > 8 mg/L; linezolid > 4 mg/L; mupirocin > 1 mg/L; rifampin > 0.016 mg/L; sulfamethoxazole > 128 mg/L; streptomycin > 16 mg/L; quinupristin-dalfopristin > 1 mg/L; tetracycline > 1 mg/L; tiamulin > 2 mg/L; trimethoprim > 2 mg/L; vancomycin > 2 mg/L. For quality control of resistance testing, the *S*. *aureus* strain ATCC 29213 and *Enterococcus faecalis* strain ATCC 29212 were used.

## 5. Conclusions

The study illustrates that linezolid-resistant MRSA are rarely found in livestock and food samples from Germany. Due to the multiple resistance to antimicrobials these strains might cause difficult-to-treat infections in animals or humans. Plasmid-coded linezolid resistance might be transmitted to other staphylococcal species or even to other genera such as *Enterococcus*. Since linezolid is not licensed for use in livestock farming, co-selection by other antibiotics such as florfenicol might take place. LR-MRSA from livestock might be transmitted to humans by direct or indirect contact. Therefore, even if rarely found at the moment, it is important for human health to monitor the possible reservoirs for bacteria with resistance to critically important antimicrobials.

## Data Availability

The assembled sequences of the LR-MRSA strains in this study are deposited in NCBI under the BioProject ID PRJNA634452.
